# Tuberculous Pancreatic and Liver Abscesses Presenting as Obstructive Jaundice in an Immunocompetent Patient

**DOI:** 10.7759/cureus.18565

**Published:** 2021-10-07

**Authors:** Eihab A Subahi, Ali B Mahgoub, Khaldun Obeidat, Ahmed M Elamin, Fatima A Rasoul

**Affiliations:** 1 Internal Medicine Department, Hamad Medical Corporation, Doha, QAT

**Keywords:** anti-tubercular therapy (att), abdominal tuberculosis, intra-abdominal abscess, liver abscess, pancreatic abscess

## Abstract

Isolated hepatobiliary, pancreatic, and peripancreatic tuberculosis (TB) is an extremely rare disease, particularly in immunocompetent individuals. To the best of our knowledge, the presentation of combined pancreatic and liver abscesses is a particularly uncommon presentation among the reported cases in the literature. This presents a significant challenge in clinical diagnosis. In this report, we discuss the case of a 24-year-old Nepalese man who presented with epigastric pain and jaundice. Further, tuberculous pancreatic and liver abscesses were detected by abdominal CT and MRI, which were later confirmed by endoscopic ultrasound-guided fine-needle aspiration (EUS-FNA) with pus smear and polymerase chain reaction (PCR) test. These tests were positive for acid-fast bacilli (*Mycobacterium tuberculosis*). The patient responded well to anti-tubercular therapy (ATT). As the clinical presentation is often unclear and radiological imaging may be misleading, physicians should have a high index of suspicion for TB, especially if the patient is young and belongs to an area highly endemic for TB.

## Introduction

Abdominal tuberculosis (TB) is a very rare clinical condition, despite the high prevalence of TB worldwide [1,2,3]. it most commonly affects the gastrointestinal tract, lymph nodes, peritoneum, and other organs in different compositions. Approximately two-thirds of patients with abdominal TB have abdominal lymphadenopathy or peritoneal disease along with intestinal involvement. The remaining one-third may also have extra-intestinal involvement [3,4,5]. Isolated hepatobiliary or pancreatic and peripancreatic TB is uncommon, particularly in immunocompetent individuals [1,2,3,6]. It usually occurs in the background of miliary TB or a widely disseminated disease [2,7]. Pancreatic TB has a different presentation. It may manifest as a pancreatic abscess, acute or chronic pancreatitis, or pancreatic mass [2,6,7,8]. Clinically and radiologically, pancreatic TB can behave as a pancreatic malignancy [6,8]. Consequently, most cases of pancreatic TB are diagnosed after surgery. However, thanks to the recent advances in modern imaging modalities, such as endoscopic ultrasound-guided fine-needle aspiration (EUS-FNA), preoperative diagnosis of pancreatic masses is feasible without surgery [2]. EUS-FNA can be used both to obtain tissue samples for cultures, staining, and polymerase chain reaction (PCR), as well as to perform the aspiration of the abscess [9,10]. The disease diagnosis can be delayed because of the nonspecific clinical presentation; however, once diagnosed, it responds favorably to anti-tubercular therapy (ATT). Most of the available data related to hepatobiliary or pancreatic TB is predominantly from case reports or series [3,6,8]. In this report, we present a case of a rare form of abdominal TB, which presented with pancreatic and liver abscesses and was finally diagnosed and confirmed by radiological images and EUS-FNA. Our main objective is to bring the attention of the physicians to this rare presentation so that they will include it as a differential diagnosis, especially for young patients hailing from areas highly endemic for TB.

## Case presentation

The patient was a 24-year-old Nepalese man with no past medical history. He presented to our emergency department (ED) with a two-month history of epigastric pain. The pain was dull in nature, intermittent, moderate in severity, radiating to his back, and sometimes increased by food intake. Ten days before his presentation to ED, he had started to have a fever, sometimes associated with chills and yellowish discoloration of his eyes. He also reported a 10-kg weight loss in the last two months, accompanied by a loss of appetite. He worked as a cleaner in a hotel and lived in shared accommodation; however, he had no history of contact with a sick person.

On presentation, he was febrile with low-grade fever, and other vital signs were stable. He was jaundiced but not pale or cyanosed on examination, with no signs of chronic liver disease. His abdomen was soft with mild epigastric tenderness. No hepatosplenomegaly, palpable masses, or flank tenderness were noted. Examination of the other systems was also unremarkable. Laboratory findings on admission showed unremarkable leukocyte count, platelet, and hemoglobin. The coagulation profile was within normal limits. Urea and creatinine levels were normal. His total bilirubin was high (62 µmol/L), and direct bilirubin was 48 µmol/L; the other parameters were as follows - alkaline phosphatase: 234 U/L; alanine transaminase: 115 U/L; and aspartate transaminase: 86 U/L. Albumin levels were low (30 g/L), amylase levels were high (87 U/L), and lipase levels were normal. The C-reactive protein level was high (102 mg/L). His interferon-γ release assay (QuantiFERON-TB Gold) test results were positive. Both the human immunodeficiency virus and viral serology (hepatitis A, B, C, E, and non-hepatotropic virus) tests were negative. The blood cultures showed no growth. The tumor markers CA 19-9 and alpha-fetoprotein (AFP) were normal.

**Figure 1:**
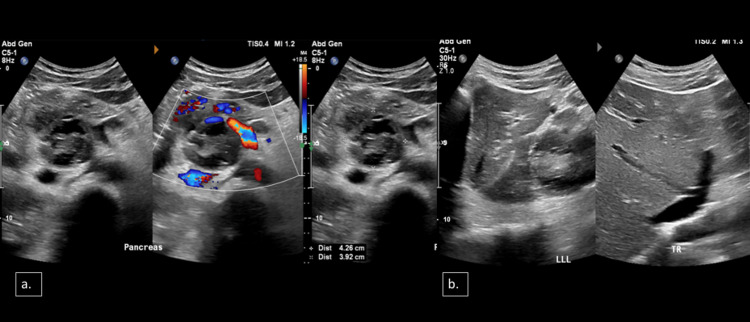
Selected axial ultrasound images of the upper abdomen (a) A heterogeneously echoic lesion with multiple cystic areas was noted in the region of the pancreatic head with peripheral vascularity (4.3 × 3.9 cm). (b) A mild degree of intrahepatic biliary radicles

His chest radiograph was unremarkable. Initially, his abdominal ultrasound showed a heteroechoic cystic lesion measuring 4.3 × 3.9 cm in the pancreatic head with peripheral vascularity (Figure 1a). The liver showed a coarse echotexture. Mild intrahepatic biliary radical dilatation was noted in the left lobe (Figure 1b). No focal lesion was noted. The common bile duct (CBD) was dilated, measuring 9.9 mm. No obvious stones were imaged in the visualized parts of the CBD.

MRI of the abdomen and magnetic resonance cholangiopancreatography (MRCP) showed relatively a well-defined lobulated heterogeneous and hyperintense necrotic lesion. This lesion involved the pancreatic head and caudate lobe of the liver, displacing the adjacent structures. It showed a peripheral thin enhancing and hypointense rim, and some internal enhancing septations with diffusion restriction of its central fluid contents and wall were noted. This most likely represented a large abdominal cold abscess arising from a conglomerate necrotic peripancreatic/portacaval lymph nodal mass, which had further extended to the pancreatic head and caudate lobe of the liver. Two small adjacent peripherally enhancing cystic lesions in segment VIII of the liver appeared bright on diffusion-weighted imaging, likely representing microabscesses. The necrotic abscess compressed the CBD and caused moderate upstream CBD (approximately 13 mm) and mild intrahepatic duct dilatation. The radiologist suggested TB infection as the first diagnosis.

**Figure 2:**
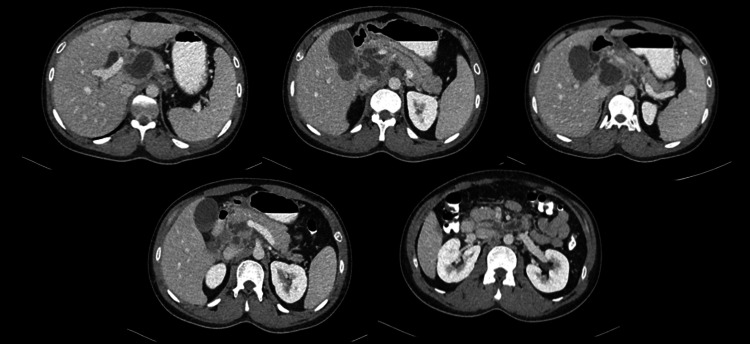
Axial images of contrast-enhanced CT of the abdomen A well-defined lobulated hypodense lesion with rim enhancement involving the pancreatic head, caudate lobe, and peripancreatic lymph nodes exerting mass effect seen as dilatation of the hepatic ducts, common bile duct, and pancreatic duct as well as compression of the portal vein and inferior vena cava with no thrombus formation CT: computed tomography

CT showed redemonstrations of lesions that were seen on MRI, which involved mainly the hepatic caudate lobe extending to the porta hepatis and peripancreatic and pancreatic head, suggestive of an abscess (tuberculous), with other adjacent necrotic lymph nodes in the upper abdomen. Tiny hepatic lesions in segment VIII were probably microabscesses and inflammatory changes in segment VI. Mass effect on the CBD (with upstream dilatation) and portal vein (Figure 2) was noted. The chest CT scan was unremarkable.

The patient underwent EUS with upper gastrointestinal endoscopy for the aspiration of the abscess and the establishment of a definitive diagnosis. EUS showed that the intrahepatic bile ducts were dilated. There were multiple large lymph nodes with anechoic areas inside (necrotic lymph nodes) in the liver hilum and para-aortic and para-duodenal areas.

The pancreatic parenchyma had diffused stranding and hyperechogenic foci. A cystic mass lesion measuring 40 × 40 mm was observed between the liver hilum and pancreatic head. This cystic mass had internal echogenicity and was in contact with the liver parenchyma and pancreatic head. The overall clinical picture was compatible with an abscess. The CBD was normal; however, it was not possible to follow the CBD owing to the compression of the cystic mass. The CHD was slightly dilated. FNA of the abscesses was performed using a 19-gauge FNA needle. Approximately 20 mL of gray-yellowish pus was aspirated from the abscess. TB culture, TB PCR, AFP staining, cytopathology, and bacterial culture were performed. Later on, his fluid smear for acid-fast bacilli (AFB) and PCR was positive for *Mycobacterium tuberculosis*, and the fluid culture showed scanty growth of *Stenotrophomonas maltophilia*, which was treated with trimethoprim-sulfamethoxazole for seven days. The diagnosis of pancreatic and liver tuberculous abscesses was established based on the above findings and results; subsequently, the patient was treated with ATT, which included isoniazid, rifampicin, ethambutol, and pyrazinamide. The patient showed a good clinical response to ATT.

## Discussion

TB is a multi-organ bacterial disease caused by different strains of *Mycobacteria*, the most common being *Mycobacterium tuberculosis*. Pulmonary TB is the most frequent clinical presentation of TB, whereas extrapulmonary TB represents almost 20% of all cases of TB in immunocompetent individuals, in which abdominal TB represents 12% of the cases, especially in patients with military TB [11,12,13]. Extrapulmonary TB is characterized by the presence of TB at sites other than the lungs. It can involve almost any organ, with the most usual sites of infection being the lymph nodes, pleura, genitourinary tract, and bones [11,13]. Abdominal TB is the sixth most frequent presentation of extrapulmonary TB and includes infections that occur in the gastrointestinal tract, peritoneum, and other abdominal organs, such as the liver and pancreas [13].

Abdominal TB, especially pancreatic and hepatobiliary variants, usually manifests gradually, with nonspecific symptoms [6,8,11]. In a study by Saluja et al., the three most frequent presenting symptoms in patients with pancreatic TB were abdominal pain, jaundice, and weight loss [4,8]. Other clinical manifestations may include fever, gastrointestinal bleeding as a result of splenic vein thrombosis, and anorexia [4,8]. If a diagnosis of pancreatic TB is suspected, initial investigations, such as tuberculin skin testing and an interferon-γ release assay, may be negative in such individuals. Sharma et al. suggested that the sensitivity of tuberculin skin testing in individuals with abdominal TB may fall between 58% and 100% [11]. With the broad range of sensitivities of TB screening tests and almost vague and diverse clinical scenarios of pancreatic and hepatobiliary TB, the diagnosis depends to a great degree on radiological and histopathological findings. In our case, the patient presented with abdominal pain, mainly epigastric, associated with fever, jaundice, and weight loss. His interferon-γ release assay for TB (QuantiFERON-TB Gold) was positive from the beginning.

Ultrasonography or CT is the diagnostic modality of choice for diagnosing individuals presenting with signs of pancreatic disease [2,11]. Ultrasound is usually the first modality used for the diagnosis of pancreatic TB, which may show a focal hypoechoic mass or cystic lesion of the pancreas.

CT is considered the investigation of choice for pancreatic disease. CT scan may show a hypodense lesion with irregular borders in the head of the pancreas, diffuse enlargement of the pancreas, or enlarged peripancreatic lymph nodes [3,14,15]. MRI findings of focal pancreatic TB include a sharply demarcated mass lesion in the pancreatic head showing heterogeneous enhancement [3,16]. In our case, abdominal ultrasound showed a heteroechoic cystic lesion in the pancreatic head, and CT showed diffuse stranding and hyperechogenic foci in the pancreatic parenchyma and a relatively well-defined lobulated heterogeneous T2 hyperintense necrotic lesion involving the pancreatic head and the caudate lobe of the liver. Several imaging modalities are used for pancreatic biopsy, including CT or ultrasound-guided percutaneous biopsy or EUS-FNA [2]. The American Joint Commission on Cancer recommends EUS-FNA as the diagnostic investigation of choice in individuals with pancreatic masses and has found it to be the most sensitive and specific technique for identifying pancreatic masses' etiology [2]. AFB is usually not recognized with FNA. In a study by Farar et al., almost 40% of individuals with abdominal TB were negative for AFB [17]. Physicians should be aware of the relatively low yield of FNA specimens to detect AFB. Therefore, culture is needed to find evidence of *Mycobacterium tuberculosis* [13,18]. In this case, the patient underwent EUS-FNA to confirm the diagnosis and sample collection. His AFP smear and PCR results were positive for AFB (*Mycobacterium tuberculosis*).

Once the diagnosis of abdominal TB is established, anti-TB therapy may be effective in treating this infection. At least six months of anti-TB therapy is usually indicated to achieve complete recovery of pancreatic lesions and resolution of symptoms. After treatment with anti-TB therapy, follow-up CT imaging may show the complete disappearance of tuberculous pancreatic lesions and may help physicians determine the treatment course duration [15].

## Conclusions

Hepatobiliary, peripancreatic, and pancreatic tuberculous abscesses are extremely rare. As the clinical presentation is often unclear and radiological imaging may be similar to that of malignancy, physicians should maintain a high index of suspicion for TB, especially in young patients belonging to areas highly endemic for TB. The application of EUS-FNA to obtain pathological evidence is extremely important to reach a correct diagnosis. Once the diagnosis is established, ATT may be sufficient to relieve the symptoms and achieve possible abscess resolution.

## References

[1] Raghavan P, Rajan D (2012). Isolated pancreatic tuberculosis mimicking malignancy in an immunocompetent host. Case Rep Med.

[2] Kaushik N, Schoedel K, McGrath K (2006). Isolated pancreatic tuberculosis diagnosed by endoscopic ultrasound-guided fine needle aspiration: a case report. JOP.

[3] Xia F, Poon RT, Wang SG, Bie P, Huang XQ, Dong JH (2003). Tuberculosis of pancreas and peripancreatic lymph nodes in immunocompetent patients: experience from China. World J Gastroenterol.

[4] Saluja SS, Ray S, Pal S, Kukeraja M, Srivastava DN, Sahni P, Chattopadhyay TK (2007). Hepatobiliary and pancreatic tuberculosis: a two decade experience. BMC Surg.

[5] Leder RA, Low VHS (1995). Tuberculosis of the abdomen. Radiol Clin North Am.

[6] Hari S, Seith A, Srivastava DN, Makharia G, Pal S (2005). Isolated tuberculosis of the pancreas diagnosed with needle aspiration: a case report and review of the literature. Trop Gastroenterol.

[7] Bakhshi G, Bhattu A (2008). Primary pancreatic tuberculosis: a case report. Bombay Hosp J.

[8] D'Cruz S, Sachdev A, Kaur L, Handa U, Bhalla A, Lehl SS (2003). Fine needle aspiration diagnosis of isolated pancreatic tuberculosis. A case report and review of literature. JOP.

[9] Song TJ, Lee SS, Park DH (2009). Yield of EUS-guided FNA on the diagnosis of pancreatic/peripancreatic tuberculosis. Gastrointest Endosc.

[10] Chatterjee S, Schmid ML, Anderson K, Oppong KW (2012). Tuberculosis and the pancreas: a diagnostic challenge solved by endoscopic ultrasound. A case series. J Gastrointestin Liver Dis.

[11] Sharma SK, Mohan A (2004). Extrapulmonary tuberculosis. Indian J Med Res.

[12] Haddad FS, Ghossain A, Sawaya E, Nelson AR (1987). Abdominal tuberculosis. Dis Colon Rectum.

[13] Rathi P, Gambhire P (2016). Abdominal tuberculosis. J Assoc Physicians India.

[14] Takhtani D, Gupta S, Suman K, Kakkar N, Challa S, Wig JD, Suri S (1996). Radiology of pancreatic tuberculosis: a report of three cases. Am J Gastroenterol.

[15] Nagar AM, Raut AA, Morani AC, Sanghvi DA, Desai CS, Thapar VB (2009). Pancreatic tuberculosis: a clinical and imaging review of 32 cases. J Comput Assist Tomogr.

[16] De Backer AI, Mortelé KJ, Bomans P, De Keulenaer BL, Vanschoubroeck IJ, Kockx MM (2005). Tuberculosis of the pancreas: MRI features. AJR Am J Roentgenol.

[17] Farer LS, Lowell AM, Meador MP (1979). Extrapulmonary tuberculosis in the United States. Am J Epidemiol.

[18] Levine R, Tenner S, Steinberg W, Ginsberg A, Borum M, Huntington D (1992). Tuberculous abscess of the pancreas. Case report and review of the literature. Dig Dis Sci.

